# Assessment of psychological personality traits in transgender groups using the Minnesota multiphasic personality inventory

**DOI:** 10.3389/fpsyg.2024.1416011

**Published:** 2024-06-12

**Authors:** Yan Liu, Zhanqiang Wang, Hanwen Dong, Yueqian Zhang, Kebing Yang, Qingyan Yang, Zhiren Wang, Yajuan Niu

**Affiliations:** ^1^Beijing Huilongguan Hospital, Beijing, China; ^2^Chengde Medical University, Chengde, Hebei, China

**Keywords:** transgender, MMPI, personality psychological characteristics, psychological assessment, China

## Abstract

**Objective:**

To explore the psychological personality characteristics of transgender groups and to determine whether these characteristics differ according to sociodemographic factors.

**Methods:**

This cross-sectional study was conducted between January 2021 and April 2023 at a psychosexual outpatient clinic in a psychiatric hospital in Beijing, China. In total, 481 individuals were included in this study, and demographic information was collected using a self-administered general questionnaire. Psychological personality traits were assessed using the Minnesota Multiphasic Personality Inventory (MMPI).

**Results:**

The mean scores of the assigned male at birth (AMAB) group were significantly higher than those of the male controls for all 10 clinical factors of the MMPI (*p* < 0.01 or *p* < 0.001). The scores for both the Masculinity-femininity (Mf) and Depression (D) factors in the AMABs group exceeded the clinical threshold (*T* > 60) and were the highest and second-highest scores on the entire scale, respectively. Individuals assigned female at birth (AFAB) had significantly higher scores than female controls for Hysteria (Hy), Psychopathic Deviate (Pd), and Hypomania (Ma) (*p* < 0.05, *p* < 0.01, and *p* < 0.001, respectively). There were significant differences in the rates of abnormal values for the various factors of the MMPI (*T* > 60) according to gender, age, and education (*p* < 0.05, *p* < 0.01, and *p* < 0.001, respectively). Compared to AFABs, AMABs had higher rates of abnormal scores (*T* > 60) on the Hypochondriasis (Hs), D, Hy, Mf, Paranoia (Pa), Psychasthenia (Pt), Schizophrenia (Sc), and Social Introversion (Si) scales (*p* < 0.05, *p* < 0.01, and *p* < 0.001, respectively). Second, the transgender group aged ≤25 years had higher rates of abnormal scores (*T* > 60) on the Hs, D, Hy, Pd, Pa, Pt, Sc, and Ma scales (*p* < 0.05, *p* < 0.01, and *p* < 0.001, respectively). Finally, outliers (*T* > 60) for the Hs, D, Hy, Pd, Pa, Pt, Ma, and Si factors were more prevalent among those with a primary to high school level of education (*p* < 0.05, *p* < 0.01, and *p* < 0.001, respectively).

**Conclusion:**

Assigned male at births may have a variety of psychological vulnerabilities, and there is a need to focus especially on those with a primary to high school level of education, those aged ≤25 years, and transgender females.

## Introduction

“Transgender” is a term that refers to individuals whose gender identity or gender expression differs from the gender assigned at birth, and it refers to both individuals assigned male at birth (AMAB) and those assigned female at birth (AFAB) (Davey et al., 2014). These individuals have a strong and persistent desire to obtain a gender opposite to their current biological one, with some often feeling uncomfortable with their physiological gender and seeking to undergo gender reassignment surgery based on a formal diagnosis from a psychiatrist (Davidson, 2007). Approximately 0.1–0.5% of the global population identifies as transgender (Winter et al., 2016). A study from the United States showed that before starting gender confirmation treatment, transgender individuals did not differ in physiological indicators such as height, weight, or blood pressure compared to the general population of the same age (Millington et al., 2020). Research also shows that there is no difference in brain capacity between transgender individuals and the general population, although transgender women were found to have thicker inferior temporal gyrus cortices (Xerxa et al., 2023). As a sexual minority, the transgender community experiences societal discrimination and prejudice, leading to heightened stress that severely affects their mental health, as well as psychological problems such as depression and anxiety (Xia and Liu, 2021).

The *Standards of Care for the Health of Transsexual, Transgender, and Gender Nonconforming People (version 7)* recommends that mental health professionals should screen for psychopathologies during the gender transition period and address identified issues as part of the overall treatment plan to provide the most appropriate healthcare for the transgender population (Cavanaugh et al., 2016). The Minnesota Multiphasic Personality Inventory (MMPI) is currently one of the most widely used self-report questionnaires in psychological diagnosis and assessment. It covers a wide range of personality traits and psychological symptoms, and its unified testing method and scoring criteria ensure that the results of different testers can be compared. Many Chinese and international studies have used various personality assessment scales to understand the personality and psychological characteristics of transgender groups, but the results vary (Cui et al., 1998; Miach et al., 2000; Li et al., 2005; Bonierbale et al., 2016; Zhang et al., 2022b). In Li Site’s study, 10 clinical scale scores of 32 transgender males were higher than those of the control group (Cavanaugh et al., 2016), while in Zhang Fei’s study, only the average *T*-score of the Masculinity-femininity (Mf) subscale was higher than that of the control group (Zhang et al., 2022b). A study from France showed significant differences in T-scores between transgender males and transgender females on four clinical scales (hypochondria, depression, hysteria, and paranoia) (Bonierbale et al., 2016). Some researchers believe that transsexual patients may have various psychological disorders (Cui et al., 1998), while others suggest that transsexuality is associated with severe personality disorders (Miach et al., 2000). Discrepancies in results among previous studies may be related to the small sample sizes and regional and cultural differences (Zhang et al., 2022a), and there has been relatively little research on the relationships of psychological function with gender, age, and education in transgender groups.

Therefore, we hypothesized that the multiple personality traits of Chinese transgender groups may differ from the general population and that these traits may vary by physiological gender, age, and education. To address this gap, this study enrolled a large Chinese transgender population to evaluate the personality characteristics of transgender individuals seeking psychiatric psychological treatment. We compared the personality traits of transgender individuals with differing physiological genders, aiming to expand research on transgender groups to various cultural and social contexts.

## Methods

### Subjects

This study collected data from 511 patients who attended the sexual psychology outpatient clinic of a specialized mental hospital in Beijing from January 2021 to April 2023. All patients presented with the main complaint of gender incongruity. The inclusion criteria were: ① Meeting the International Classification of Diseases, 10th Revision (ICD-10) diagnostic criteria for “transsexualism”; ② having at least a junior high school level of education and being over 16 years old; ③ not having undergone gender reassignment surgery; ④ Voluntarily participating in the study and signing an informed consent form, and ⑤ not having severe cognitive impairment and being capable of independently completing the test. Exclusion criteria were: ① Transvestic fetishism; ② dependence on or abuse of psychoactive substances; ③ Asperger’s syndrome, related spectrum disorders, etc.; ④ non-cooperation with or drop out during the study; and ⑤ a score > 10 points on the Lie scale (L). A total of 481 transgender individuals met the study criteria, of whom 405 were AMABs and 76 were AFABs; 384 were aged 25 years old and under and 97 were aged over 25 years; 132 had a primary to high school level education (junior high school, senior high school), and 349 received higher education (university, postgraduate).

To obtain rigorous control data, this study did not collect a control group but instead used the Chinese general population norms reported by Song (1985).

### Procedure

Patients were evaluated and treated in the sexual psychology clinic of a psychiatric hospital in Beijing according to the nursing standards of the World Transgender Health Association. At least two psychiatrists evaluated whether patients met the diagnostic criteria for “susceptibility” in the International Classification of Diseases Tenth Revision (ICD-10), and all patients meeting those criteria were aged ≥16 years. All data analyzed in this study were collected during the diagnostic phase. The MMPI data were collected by three professionally qualified psychological assessors.

### Tools

#### General information

During each consultation, the physician conducted an interview and asked patients about their physiological sex, age, educational level, marital status, and other general demographic information.

#### Minnesota multiphasic personality inventory

This study used the Chinese version of the MMPI compiled by the Institute of Psychology of the Chinese Academy of Sciences. The reliability and validity of the Chinese version of the MMPI have been confirmed in previous studies (Song, 1985). It includes 10 clinical scales [Hypochondriasis (Hs), Depression (D), Hysteria (Hy), Psychopathic Deviate (Pd), Mf, Paranoia (Pa), Psychasthenia (Pt), Schizophrenia (Sc), Hypomania (Ma), and Social Introversion (Si) scales] and three validity scales [L, Infrequency scale (F), and Defensiveness scale (K)]. Based on the actual situation in China, *T* > 60 points was considered abnormal, and the respondents completed the questionnaire survey within 45–90 min.

### Statistical analysis

This study utilized IBM SPSS 26.0 software (IBM Corp., Armonk, NY, United States) for data processing. The Kolmogorov–Smirnov test was applied to the initial test data, revealing that in the K-S test, the *p* values for the normality test of MMPI data were all greater than 0.05, indicating that the data are normally distributed. Quantitative data are presented as mean ± standard deviation, while qualitative data are reported as frequencies. An independent sample *t*-test was employed for intergroup comparisons; a *p*-value of less than 0.05 was considered statistically significant. Additionally, Excel software (Microsoft Corp., Armonk, NY, United States) was used to calculate and graph the mean *T*-scores on the MMPI subscales for transgender women, transgender men, and the general male and female populations. The chi-square test or Fisher’s exact test was utilized to compare MMPI scores between gender groups [assigned male at birth (AMAB) vs. assigned female at birth (AFAB)], age groups (>25 vs. ≤25 years), and educational groups (>high school vs. ≤high school). Independent-sample *t*-tests were conducted to compare the MMPI factor scores between transgender males and females and to compare these scores with those of the corresponding general population control groups.

## Results

There was no significant difference in age or educational level between the transgender female group and the transgender male group (*p* > 0.05).

### Comparison of mean scores and T-scores on MMPI factors between AMABs and the Chinese male norm group

As shown in Table 1, the average scores of AMABs on the 10 clinical scales of the MMPI were significantly higher than those of the male norm group (*p* < 0.01 or *p* < 0.001).

**Table 1:** Comparison of the mean and *T* scores of MMPI between AMABs and male control group.

Variables	AMABs (*n* = 405)		Male control group (*n* = 1,553)		*t*
	*x* ± *s*	*T* score	*x* ± *s*	*T* score	
L	5.34 ± 2.422	48.40	5.7 ± 2.5	51	−2.986^**^
F	15.21 ± 7.088	51.86	13.7 ± 6.9	50	4.280^***^
K	11.81 ± 4.950	47.14	13 ± 4.5	50	−4.848^***^
Hs	11.63 ± 6.865	55.47	8.8 ± 4.8	44	8.310^***^
D	31.86 ± 7.510	61.27	26.2 ± 4.9	49	15.171^***^
Hy	27.08 ± 6.354	59.05	22.0 ± 5.4	49	16.078^***^
Pd	22.87 ± 5.954	58.48	18.9 ± 4.4	50	13.424^***^
Mf	37.97 ± 15.930	72.46	27.6 ± 4.0	48	13.095^***^
Pa	14.60 ± 4.614	53.97	12.8 ± 3.9	36	7.851^***^
Pt	25.11 ± 11.429	58.75	17.9 ± 7.9	50	12.689^***^
Sc	30.20 ± 14.170	56.96	23 ± 10.2	49	10.229^***^
Ma	19.24 ± 5.480	50.89	18.5 ± 5.3	50	2.725^**^
Si	38.66 ± 9.864	55.87	34.5 ± 6.9	50	8.481^***^

MMPI, Minnesota Multiphasic Personality Inventory; AMAB, Assigned male at birth; T-score is the exported score of the original score; Hs, Hypochondriasis; D, Depression; Hy, Hysteria; Pd, PsychopathicDeviate; Mf, Masculinity-femininity; Pa, Paranoia; Pt, Psychasthenia; Sc, Schizophrenia; Ma, Hypomania; Si, Social Introversion; ^*^p < 0.05; ^**^p < 0.01; and ^***^p < 0.001.

### Comparison of mean scores and T-scores on MMPI factors between AFABs and Chinese female norm group

As shown in Table 2, the scores of AFABs on the Hy, Pd, and Ma scales were significantly higher than those of the national female norm group (*p* < 0.05, *p* < 0.01, or *p* < 0.001), whereas their scores on the Mf and Si scales were significantly lower than those of the national female norm group (*p* < 0.001). None of the scores on the scales exceeded the clinical thresholds.

**Table 2:** Comparison of the mean and *T* scores of MMPI between AFABs and female control group.

Variables	AFABs (*n* = 76)		Female control group (*n* = 1,516)		*t*
	*x* ± *s*	*T* score	*x* ± *s*	*T* score	
L	5.47 ± 2.403	49.14	5.7 ± 2.5	51	−0.821
F	11.71 ± 6.866	49.74	11.7 ± 5	50	0.013
K	13.74 ± 4.867	52.84	12.3 ± 4.3	49	2.574^*^
Hs	9.58 ± 7.478	48.79	9.8 ± 4.9	50	−0.258
D	27.41 ± 7.586	47.84	28.4 ± 5.0	49	−1.140
Hy	24.43 ± 6.814	52.18	22.8 ± 5.5	50	2.091^*^
Pd	20.71 ± 5.812	54.97	18.3 ± 4.5	49	3.616^**^
Mf	29.41 ± 5.141	54.91	31.8 ± 3.9	51	−4.056^***^
Pa	12.32 ± 3.977	48.66	12.8 ± 3.9	50	−1.061
Pt	18.67 ± 11.132	49.58	18.8 ± 7.8	50	−0.101
Sc	23.05 ± 14.043	50.26	22.5 ± 9.6	50	0.343
Ma	19.42 ± 5.512	54.89	16.6 ± 5.2	50	4.462^***^
Si	32.30 ± 8.890	43.14	37.3 ± 6.7	49	−4.900^***^

MMPI, Minnesota Multiphasic Personality Inventory; AFAB, Assigned female at birth; *T*-score is the exported score of the original score; Hs, Hypochondriasis; D, Depression; Hy, Hysteria; Pd, Psychopathic Deviate; Mf, Masculinity-femininity; Pa, Paranoia; Pt, Psychasthenia; Sc, Schizophrenia; Ma, Hypomania; Si, Social introversion; ^*^p < 0.05; ^**^p < 0.01; ^***^p < 0.001.

### Comparison of MMPI profile between the transgender and national norm groups

**Figure 1:**
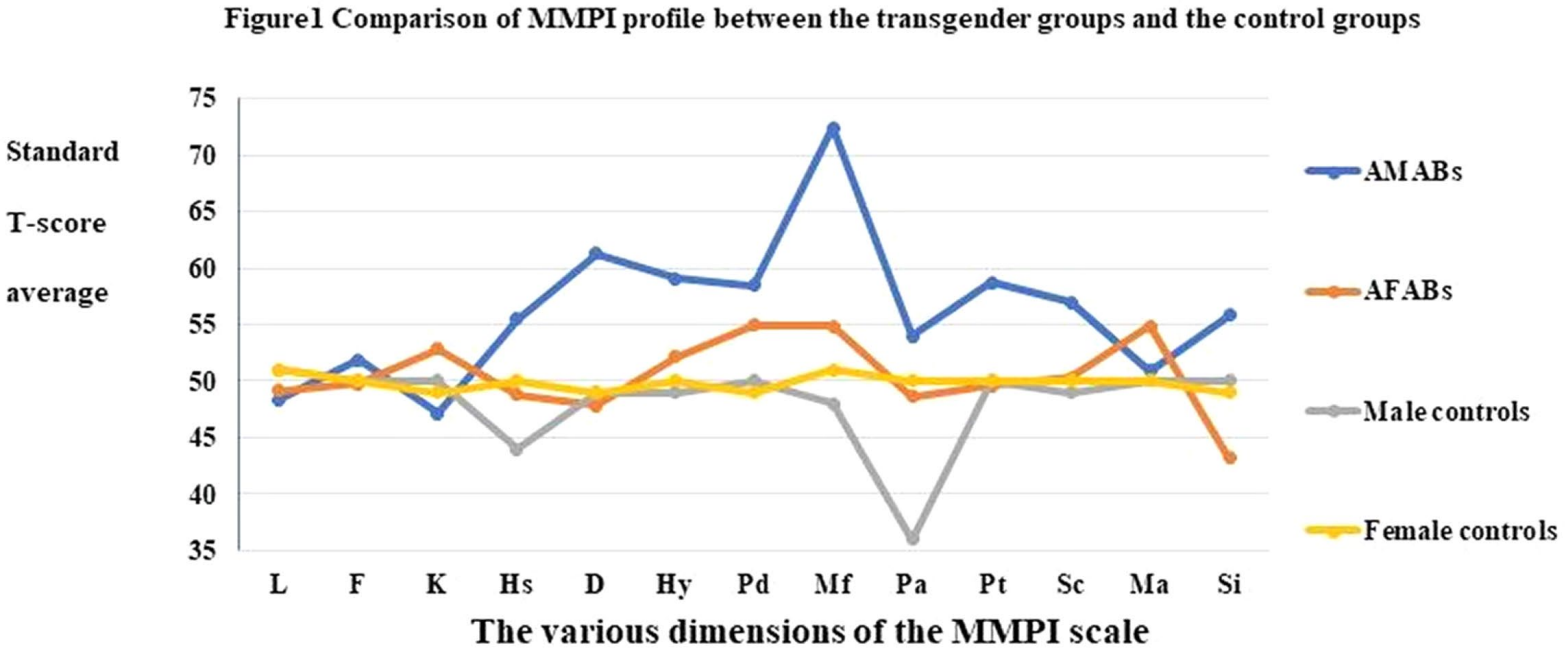
Comparison of MMPI profile between the transgender groups and the control groups.

The standard *T*-score average profiles of the AMABs, AFABs and national male and female norm groups are shown in Figure 1, with the vertical axis representing the standard *T*-score and the horizontal axis representing the MMPI scale score. The figure shows that there is a significant difference in amplitude between the profile curves of AMABs and the national male norm group. Both the Mf and D factors exceed the clinical threshold (*T* > 60) and have the highest and second-highest peaks within the entire profile. The remaining groups did not exceed the threshold on any MMPI dimensions.

### Comparison of abnormal scores (T > 60) in different MMPI dimensions according to demographic variables

As shown in Table 3, compared to AFABs, AMABs had higher rates of abnormal scores (*T* > 60) on the Hs, D, Hy, Mf, Pa, Pt, Sc, Ma, and Si scales (*p* < 0.05, *p* < 0.01, or *p* < 0.001). Second, the transgender group aged 25 years and under had higher rates of abnormal scores (*T* > 60) on the Hs, D, Hy, Pd, Pa, Pt, Sc, and Ma scales (*p* < 0.05, *p* < 0.01, or *p* < 0.001). Finally, outliers (*T* > 60) for the Hs, D, Hy, Pd, Pa, Pt, Ma, and Si factors were more prevalent among those with a primary to high school level of education (*p* < 0.05, *p* < 0.01, or *p* < 0.001).

**Table 3:** Comparison of outliers (*T* > 60) in each dimension of MMPI in different demographic variables.

Variables	Gender (number, %)		*χ* ^2^	Age (number, %)		*χ* ^2^	Education (number, %)		*χ* ^2^
	AMABs *n* = 405	AFABs *n* = 76		≤25 *n* = 384	>25 *n* = 97		Primary education *n* = 132	Higher education *n* = 349	
Hs	145 (35.8)	18 (23.7)	4.194^*^	143 (37.2)	20 (20.6)	9.549^**^	59 (44.7)	104 (29.8)	9.488^**^
D	214 (52.8)	18 (23.7)	21.785^***^	207 (53.9)	25 (25.8)	24.547^***^	76 (57.6)	156 (44.7)	6.360^*^
Hy	181 (44.7)	16 (21.1)	14.787^***^	170 (44.3)	27 (27.8)	8.651^**^	68 (51.5)	129 (37.0)	8.387^**^
Pd	185 (45.7)	29 (38.2)	1.466	181 (47.1)	33 (34.0)	5.393^*^	71 (53.8)	143 (41.0)	6.367^*^
Mf	338 (83.5)	21 (27.6)	105.347^***^	292 (76.0)	67 (69.1)	1.987	94 (71.2)	265 (75.9)	1.127
Pa	106 (26.2)	10 (13.2)	5.923^*^	104 (27.1)	12 (12.4)	9.159^**^	51 (38.6)	65 (18.6)	20.959^***^
Pt	191 (47.2)	21 (27.6)	9.901^**^	186 (48.4)	26 (26.8)	14.703^***^	78 (59.1)	134 (38.4)	16.642^***^
Sc	167 (41.2)	17 (22.4)	9.643^**^	159 (41.4)	25 (25.8)	8.012^**^	69 (52.3)	115 (33.0)	15.137
Ma	63 (15.6)	20 (26.3)	5.189^*^	74 (19.3)	9 (9.3)	5.415^*^	32 (24.2)	51 (14.6)	6.220^*^
Si	160 (39.5)	7 (9.2)	25.914^***^	140 (36.5)	27 (27.8)	2.541	55 (41.7)	112 (32.1)	3.874^*^

MMPI, Minnesota Multiphasic Personality Inventory; AMAB, Assigned male at birth; AFAB, Assigned female at birth; *T*-score is the exported score of the original score; Hs, Hypochondriasis; D, Depression; Hy, Hysteria; Pd, PsychopathicDeviate; Mf, Masculinity-femininity; Pa, Paranoia; Pt, Psychasthenia; Sc, Schizophrenia; Ma, Hypomania; Si, Social Introversion; ^*^p < 0.05; ^**^p < 0.01; ^***^p < 0.001.

## Discussion

Transgender individuals, whose gender identity and expression diverge from traditional societal, cultural, or physiological norms, are prone to prejudice and discrimination from society. This can lead to adverse emotions such as depression and anxiety, and in severe cases, to changes in personality traits. Therefore, research on the personality and psychological characteristics of the transgender population is particularly important. The MMPI, developed by Professors Hathaway and McKinley at the University of Minnesota in the 1940s, is a widely used and authoritative personality test. It is extensively applied for personality assessment and the diagnosis of mental illnesses. The revised version of this scale, introduced in China, evaluates both personality and clinical mental health, and can also be used to study the personality traits of specific populations according to their actual needs (Fang et al., 2019; Li et al., 2021; Zhu et al., 2022).

The results also show that the Mf factor score of the transgender female group was significantly higher than the clinical threshold, indicating pathology similar to previous research findings (Cui et al., 1998). This clearly reflects the huge difference between physiological and psychological genders in AMABs. However, some researchers believe that the standard scores of the MMPI’s Mf factor were derived from representative samples of cisgender individuals and suggest comparing them with data from the transgender norm group (Fleming et al., 1981). The D factor scores of the AMABs in our study also exceeded the clinical threshold, indicating that AMABs may experience clinical depression. Past research has found that one of the most common personality disorders in AMABs is depressive personality disorder, which is thought to be related to the significant stress that this group faces (Meybodi and Jolfaei, 2022). Over 75% of transgender individuals, particularly AMABs, experienced discrimination and harm related to their gender expression during their development (Peng et al., 2019). Although the scores of other factors did not exceed 60 in this study, thus indicating no clinical pathology, they were significantly higher than those of the norm group and close to the threshold for clinical significance. This suggests that the transgender female group may experience many emotional and psychological problems, and even changes in personality.

This study found that although the Hy, Pd, and Ma scale scores of AFABs were significantly higher than those of cisgender women, whereas those of the Mf and Si scales were significantly lower than those of cisgender women, the thresholds for clinical significance were not exceeded. This is inconsistent with previous research. Cui et al. (1998) found that the Pd and Mf scale scores of AFABs exceeded the threshold of 60 points, and later, these findings were replicated by Li et al. (2005). These discrepancies may be related to small sample sizes and sample distributions (Zhang et al., 2022a). We also believe that they may reflect less social pressure faced by AFABs; compared to AMABs, society is more accepting of AFABs (Shirdel-Havar et al., 2018).

To understand the risk factors for personality disorders in the transgender population, this study also explored the distribution of abnormal values on various dimensions of the MMPI according to different variables. The results indicated that AMABs are more likely to show deviations in one or more personality traits, and the personality of AFABs was found to be more stable. Other studies have also found that the functioning of AFABs is better than that of AMABs (Gómez-Gil et al., 2008). In addition to the greater pressure faced by AMABs mentioned above, these results may also be related to the fact that AFABs are more adaptable to their environment (Shirdel-Havar et al., 2018). Second, we found that the younger and less educated members of our transgender group were more likely to show deviation in one or more personality traits. This may be related to an inability to deal with gender dysphoria when it arises. However, when Annelou compared adult and adolescent transgender groups using the MMPI, they found that the proportion of abnormal Pa and Pt scale scores in the transgender adult group was higher than that in the transgender adolescent group (de Vries et al., 2011). This may be related to the age classes; how to scientifically divide and explore the risk factors for deviant personality traits in this population needs further research.

In summary, this study is the first to conduct a comprehensive analysis of the personality and psychological characteristics of the transgender population in China using a large sample. The findings suggest that individuals’ AMABs may face multiple psychological challenges and exhibit varying degrees of personality trait deviations. Clinical consultations should significantly focus on the personality and psychological development of AMABs, especially those under 25 years old or with a high school education or lower. This study broadens the scope of research on transgenderism into new cultural and social contexts. By highlighting the mental health disparities between transgender groups and the general population, it aims to enhance public understanding and support for transgender individuals, mitigate prejudice and discrimination, and foster a more inclusive and supportive environment. Additionally, the results could assist in the development and refinement of relevant policies and serve as a basis for designing tailored psychological treatment plans for transgender individuals, aiding them in managing psychological issues and improving their mental health.

However, this study also has some limitations. Firstly, the absence of an MMPI norm group for the transgender population could have led to errors in comparing this group with national average norms. Secondly, the study considered a limited set of demographic variables. Lastly, although the MMPI is rigorously validated, its nature as a self-report test may introduce some bias. Future research should aim to establish a cross-gender MMPI norm control group, investigate and analyze additional demographic variables of transgender groups, and integrate the assessment of cross-gender MMPI data with other physiological indicators, such as heart rate variability and infrared thermography.

## Data availability statement

The raw data supporting the conclusions of this article will be made available by the authors, without undue reservation.

## Ethics statement

The studies involving humans were approved by Beijing Huilongguan Hospital Ethics Committee. The studies were conducted in accordance with the local legislation and institutional requirements. The participants provided their written informed consent to participate in this study.

## Author contributions

YL: Writing – original draft, Supervision, Writing – review & editing. ZhaW: Writing – review & editing, Data curation, Formal analysis, Investigation, Methodology. HD: Writing – review & editing, Data curation, Investigation, Formal analysis, Methodology. YZ: Resources, Writing – review & editing. KY: Supervision, Writing – review & editing. YQ: Validation, Writing – review & editing. ZhiW: Funding acquisition, Writing – review & editing. YN: Formal analysis, Methodology, Project administration, Supervision, Writing – review & editing.
